# Circulating HDL and Non-HDL Associated Apolipoproteins and Breast Cancer Severity

**DOI:** 10.3390/jcm11051345

**Published:** 2022-02-28

**Authors:** Christine Bobin-Dubigeon, Hassan Nazih, Valentin Blanchard, Mikaël Croyal, Jean-Marie Bard

**Affiliations:** 1 CRNHO, West Human Nutrition Research Center, Université de Nantes, EA 2160-IUML FR3473 CNRS, F-44035 Nantes, France; christine.bobin-dubigeon@univ-nantes.fr (C.B.-D.); el-hassane.nazih@univ-nantes.fr (H.N.); 2 Institut de Cancérologie de l’Ouest, F-44805 Saint-Herblain, France; 3 Department of Medecine, Centre for Heart Lung Innovation, Providence Healthcare Research Institute, St. Paul’s Hospital, University of British Columbia, Vancouver, BC V6Z1Y6, Canada; valentin.blanchard@hli.ubc.ca; 4 CRNHO, West Human Nutrition Research Center, Université de Nantes, CNRS, INSERM, l’Institut du Thorax, F-44035 Nantes, France; mikael.croyal@univ-nantes.fr; 5 CHU Nantes, Inserm, CNRS, SFR Santé, Inserm UMS 016, CNRS UMS 3556, F-44000 Nantes, France

**Keywords:** apolipoproteins, lipoproteins, HDL, non-HDL, breast cancer, Ki-67

## Abstract

Plasma lipids are carried within lipoproteins with various apolipoprotein content. This study evaluates the interest of measuring the apolipoproteins of circulating lipoproteins in breast cancer. Patients with early-stage breast cancer (*n* = 140) were included. Tumors differed by the expression of estrogen and progesterone receptor (HR− and HR+ for negative and positive expression) and the proliferation marker Ki-67 (≤20% or ≥30%). Apolipoprotein concentrations were determined in plasma, HDL and non-HDL fractions, and results are given in mg/dL, median (25th–75th). Patients did not differ in their plasma and lipoprotein lipid concentrations. HDL apoC-I and non-HDL apoC-II were reduced (1.34 (1.02–1.80) vs. 1.61 (1.32–2.04), *p* = 0.04; 0.31 (0.18–0.65) vs. 0.63 (0.39–1.02), *p* = 0.01; respectively), in RH-/high Ki-67 patients in comparison to RH-/low Ki-67 patients, while plasma apoD and HDL apoD were higher (3.24 (2.99–4.16) vs. 3.07 (2.39–3.51), *p* = 0.04; 2.74 (2.36–3.35) vs. 2.45 (2.01–2.99), *p* = 0.04; respectively). When RH+/high Ki-67 patients were compared with RH+/low Ki-67 patients, HDL apoC-I and HDL apoC-III were higher (1.56 (1.20–1.95) vs. 1.35 (1.10–1.62), *p* = 0.02; 2.80 (2.42–3.64) vs. 2.38 (1.69–2.96), *p* = 0.02; respectively). The distribution of exchangeable apolipoproteins, such as apoC-I, apoC-II, apoC-III, apoD, between lipoproteins is linked to the severity of breast cancer.

## 1. Introduction

Among the last years, several reports evidenced that various metabolic disturbances related to obesity may be associated with an increased risk of breast cancer [1,2]. Circulating lipids may be one of the factors that relate to these metabolic abnormalities and the disease risk [3]. Clinical studies looking at the association between circulating cholesterol carried by Low-Density Lipoproteins (LDL) or High-Density Lipoproteins (HDL) with breast cancer have raised conflicting results. Nevertheless, the results of large studies seem to point towards a positive relationship between LDL and breast cancer, while HDL would rather be negatively associated with the disease [4]. Apolipoproteins play essential roles in maintaining the structural integrity and functional specificity of plasma lipoproteins. They are directly involved in various metabolic processes of lipoproteins, including secretion, prevention of premature removal from the circulation, binding with cell-surface receptors and activation of lipolytic enzymes [5]. Besides their role in lipoprotein metabolism, apolipoproteins were shown to be involved in the development of breast cancer, as nicely reviewed recently [6]. The plasma level of some apolipoproteins was related to breast cancer severity. For instance, low levels of plasma apoA-I were shown to independently predict the poor clinical outcome of patients with invasive ductal breast cancer [7]. In another study, lower apoC-I and apoC-II concentrations were found in breast cancer patients when compared to controls, while apoC-III concentration was higher [8]. Women with advanced breast cancer were shown to have higher plasma concentrations of apoD [9]. The apoE plasma concentration was also positively associated with breast cancer malignancy [10]. It is known that apo E is present in plasma in a polymorphic form. The major isoforms are apoE2, apoE3 and apoE4, leading to six phenotypes, depending on the inherited ε alleles. There are some studies in the literature suggesting a link between the presence of the ε4 allele and breast cancer [11]. Therefore, it would be of great interest to consider not only the cholesterol content of lipoproteins but also their apolipoprotein composition when studying the association of circulating lipoproteins with breast cancer. The present study was undertaken to evaluate the interest of measuring the apolipoprotein content of circulating lipoproteins in the context of breast cancer.

## 2. Materials and Methods

### 2.1. Patient Samples

This study included 140 patients with early-stage breast cancer referred to our hospital (ICO René Gauducheau, Saint-Herblain, France). Biological material was collected from our biobank, which was declared to and authorized by the French Research Ministry (Declaration Number: DC-2018-3321). This declaration includes approval by a research ethics committee (CPP: Comité de Protection des Personnes) [12]. Informed consent was obtained from patients, granting permission to use their biological specimens and clinical-pathological data for research purposes, as required by the French legislation and the French committee for the protection of human rights. Patient tumors differed by the expression of estrogen (ER) and/or progesterone (PR) receptor (HR− for ER- PR- and HR+ for ER+ and/or PR+, respectively) and the level of the proliferation marker Ki-67 (Ki-67 ≤ 20% or Ki-67 ≥ 30%). Although the cut-off value of 20% is commonly used in clinical practice to categorize patients with high or low proliferative index, the value of 30% was chosen in order to avoid the “grey zone”, which could be misleading for our analyses. Serum and EDTA plasma samples from patients were collected at the time of diagnosis and before any therapeutic intervention, were retrieved from our biobank. The distribution of these sera was as follows: 92 were obtained from HR+ patients, and 48 were obtained from HR− patients. Among the HR+ group, 41 tumors were Ki-67 ≥ 30%, and 51 tumors were Ki-67 ≤ 20%. Among the HR− group, 29 tumors were Ki-67 ≥ 30%, and 19 tumors were Ki-67 ≤ 20%.

### 2.2. Biological Analyses

Plasma cholesterol, plasma triglycerides, HDL cholesterol and LDL cholesterol concentrations were measured using enzymatic kits from Diasys, according to the manufacturer’s instructions (Grabels, France). HDL and non-HDL fractions were separated from EDTA plasma by the specific precipitation of apoB-containing lipoproteins. An MgCl_2_ solution (2 mol/L, 2.5 µL) was added to plasma samples (100 µL). Then, 10 µL of 4% phosphotungstic acid (in NaOH 1 mol/L:water; 16:84; *v*:*v*) were added. Samples were vortex-mixed and centrifuged for 30 min (4 °C, 4000× *g*). Supernatants (HDL fraction) were collected, and the pellets (non-HDL fraction) were resuspended in 100 µL of ultra-pure water. Total plasma, HDL and non-HDL apolipoproteins A-I, B100, C-I, C-II, C-III, D and E, as well as apoE phenotyping, were determined by liquid chromatography–tandem mass spectrometry, as described previously [13]. Briefly, the apolipoproteins were quantified in 40 µL sample aliquots using trypsin proteolysis and the subsequent analysis of proteotypic peptides. The intra- and inter-assay variabilities did not exceed 6.5% in plasma and HDL samples. The non-HDL concentrations were deducted by subtracting total plasma and HDL concentrations. The calculated non-HDL concentrations were compared to the direct measurement of apolipoproteins in the precipitated non-HDL fraction in a representative set of samples (25%). Since coefficients of variation did not exceed 6.8% between both measurements, we kept the calculated non-HDL values for analyses to gain sensitivity. 

### 2.3. Statistical Analyses

All statistics were calculated using SAS software (Chapel Hill, NC, USA) version 9.04. The univariate procedure was also used to determine median and 25th and 75th intervals for these variables. The statistical differences between biological variables in EDTA plasma, HDL and non-HDL between patients with different levels of proliferation were estimated by median test analyses in each subset of samples, HR+ and HR−. 

## 3. Results

Table 1 presents the clinical characteristics of the studied population. HR+ patients were significantly older (*p* = 0.003) in the group with low proliferative index, explaining the difference in menopausal status (*p* = 0.006). However, no difference in BMI and normolipidemic drug consumption were observed. Infiltrating duct carcinoma was the most common type of cancer found. As expected, the histopronostic grade of tumors differed according to the HR/Ki-67 groups with the most pejorative grade in the Ki-67 ≥ 30 both for HR+ and HR− groups (*p* < 0.001). Tumors of all HR+ patients with a high Ki-67 level were luminal B HER2- 26/41 (63.4%) or HER2+ 15/41 (36.6%), while in the sub-group of low Ki-67 level, 46/51 (90.2%) were luminal A and 5/51 (9.8%) were luminal B HER2+. For HR− patients, 21/29 patients (72.4%) were triple-negative breast cancer (TNBC) in the sub-group of high Ki-67 level, and 16/19 (84.2%) were TNBC in the sub-group of low Ki-67 level. 

Table 2 shows the concentration of plasma and lipoprotein lipids in RH- and RH+ breast cancer patients and low (Ki-67 ≤ 20%) or high (Ki-67 ≥ 30%) proliferative index. In each group of hormone receptor breast cancer patients, no significant difference was observed in any plasma or lipoprotein lipid concentrations between patients with a low proliferative index and patients with a high proliferative index.

Table 3 shows the concentrations of plasma, HDL and non-HDL apolipoproteins. When considering the plasma concentrations, no difference between low proliferative index and the high proliferative index was observed, either in RH- or RH+ patients, with the exception of apoD, which was significantly increased in RH- patients with a high Ki-67 index when compared with RH- patients with a low Ki-67 index (*p* = 0.042). The patient populations did not differ for the plasma concentrations of either apoA-I, the main carrier of HDL or apoB100, the main carrier of non-HDL lipoproteins. RH- patients were characterized by a lower concentration of HDL apo-C-I when their tumor exhibited a higher proliferative index (*p* = 0.041), while the opposite was observed for RH+ patients (*p* = 0.022). In RH+ patients, a tendency towards a higher non-HDL apo-C-I concentration (*p* = 0.060) was also observed in the case of a high Ki-67 index. RH- patients with a high Ki-67 index had a lower concentration of non-HDL apoC-II than those with a low Ki-67 index (p0.009). HDL apo-C-III was significantly increased in RH+ patients with a high proliferative index, compared with RH+ patients with a low proliferative index (*p* = 0.022). In RH- patients, HDL apoD was higher in patients with a high Ki-67 index when compared with RH- patients with a low Ki-67 index (*p* = 0.035).

In order to determine if the observed differences were related to a difference in the absolute concentration of HDL or non-HDL lipoproteins carrying a given apolipoprotein or if they were related to a relative enrichment or a relative impoverishment in this apolipoprotein, the molar ratios between these apolipoproteins and apoA-I in HDL or apoB100 in non-HDL were compared between patients with a low proliferative index and patients with a high proliferative index. These results are shown in Table 4. RH- patients were characterized by a decrease in the apoC-I-to-apoA-I ratio in HDL when Ki-67 was high compared with patients with a low Ki-67 index (*p* = 0.001). The opposite was observed in RH+ patients, with higher values of this ratio in patients with a high Ki-67 value (*p* = 0.007). In RH- patients, the apoC-II-to-apoB100 ratio in non-HDL was lower when the Ki-67 was high when compared with the low Ki-67 sub-group (*p* = 0.001). The apoC-III-to-apoA-I ratio of HDL was also lower in RH- patients with a high Ki-67 value when compared with the low Ki-67 sub-group (*p* = 0.041). In RH+ patients, the apoE-to-apoB100 ratio in non-HDL was significantly higher in the sub-group of patients with a high Ki-67 value, in comparison with patients with a low Ki-67 value (*p* = 0.022). A tendency (*p* = 0.060) towards a higher apo-C-III-to-apoA-I ratio in HDL of RH+ patients with a high Ki-67 value was also observed.

## 4. Discussion

Here, we aimed to evaluate the interest of measuring the apolipoprotein content within circulating lipoproteins in breast cancer patients. We showed that patients with breast cancer of various severity display similar concentrations of plasma and lipoprotein lipids but different concentrations of some apolipoproteins carried by HDL and non-HDL. Besides, we showed that the relationship between apolipoproteins and the disease severity differs between hormone receptor-negative and hormone receptor-positive tumors. Some previously published results indicated that some apolipoproteins might be involved in the development of breast cancer. These results were nicely summarized in a recent review [6].

Despite the fact that low levels of plasma apoA-I were shown to independently predict the poor clinical outcome of patients with invasive ductal breast cancer [7], a large study conducted on 1411 women from the AMORIS cohort with breast cancer severity known only found a modest positive association between the apoB100-to-apoA-I ratio and breast cancer severity [14]. In line with these previous results, we did not find any difference between patients with high proliferative tumor index and patients with low proliferative tumor index. This was the case for RH- as well as RH+ tumors.

In a study using serum fractionation by strong anion exchange chromatography followed by mass spectrometry analysis, lower apoC-I and apoC-II concentrations were found in breast cancer patients than controls, while apoC-III concentration was higher [8]. In a recent study comparing protein profiles between breast cancer patients and controls, it was also shown that plasma apoC-I was lower in affected patients, and further analyses identified apoC-I signature peptides able to inhibit breast cancer cell proliferation in vitro [15]. It was also suggested that elevated apoC-I levels could help distinguish triple-negative breast cancer and non-triple negative breast cancer [16]. These results pointed out the potential of apoC-I as a biomarker of breast cancer. However, the design and goals of the present study were different from those published previously. Our results suggest that it may be of interest to distinguish between HDL and non-HDL apolipoproteins when studying the relationship of apolipoproteins with breast cancer. As a matter of fact, in the present study, plasma apoC-I, apoC-II and apoC-III concentrations did not differ between patients with high proliferative index tumors and patients with low proliferative index tumors. However, HDL apoC-I was lower in the case of high proliferative index tumors when breast cancer was RH-, while it was higher in high proliferative index tumors when breast cancer was RH+. HDL apoC-III was also higher in RH+ patients with a high proliferative index tumor. This deeply suggests that the relationship between apoC-I or apoC-III and breast cancer tumor behavior concerns only HDL and that it differs from the nature of the tumor. The only difference observed for apoC-II was for non-HDL concentration, which was lower in case of high proliferative index RH- tumors. The analysis of the ratios between one apolipoprotein and apoA-I for HDL or one apolipoprotein and apoB100 for non-HDL may be used to determine if these differences are related to a change in the absolute number of lipoprotein particles containing this apolipoprotein or if this is related to a change in the number of copies of a given apolipoprotein per lipoprotein particle. If this ratio increases, this means that the number of copies of this apolipoprotein per lipoprotein particle increases; if this ratio decreases, this means that the number of copies of this apolipoprotein per lipoprotein particle decreases. This change in the apolipoprotein composition of the lipoprotein particle may be independent of its absolute concentration in plasma but it reflects a modification of its quality, which may affect its biological behavior. Concerning apolipoproteins C-I, C-II and C-III, our results suggest that these differences are due to a change in the number of copies of each apolipoprotein per lipoprotein particle.

ApoD is probably one of the apolipoproteins that were the most extensively studied in the context of breast cancer. However, its interaction with the disease is rather complex. While apoD has been shown to inhibit the proliferation of breast cancer cells [17], estrogens significantly reduce apoD gene expression [18]. At the cell level, apoD may influence several critical pathways, including MAPK, 5-LO and COX-2 pathways [19,20,21,22]. It was also suggested that apoD expression could be predictive of breast cancer recurrence in tamoxifen-treated patients [23,24,25,26,27,28]. However, a large population-based case-control study on 11,251 women with well-characterized tumors failed to demonstrate any association between apoD nuclear and cytoplasmic expression and disease recurrence in HR− as well as tamoxifen-treated HR+ patients [29]. Nevertheless, women with advanced breast cancer were shown to have higher plasma concentrations of apoD [9], and apoD was suggested to be a good prognostic indicator for the disease [30]. In the present study, we showed that plasma apoD is higher in the case of high proliferative index tumors, only in the case of RH- breast cancer tumors. In addition, this difference was observed for HDL only, while non-HDL apoD did not differ with the proliferative index of tumors. However, when calculating the ratio between apoD and apoA-I in HDL, these differences disappeared, suggesting that the relationship between HDL apoD and RH- breast cancer severity is due to an increased number of HDL particles carrying apoD, and not to a relative enrichment of HDL in apoD.

On the cellular level, apoE may inhibit angiogenesis and the proliferation of breast cancer cells [31,32]. By contrast, in a clinical study, it has been shown that the apoE plasma concentration is positively associated with breast cancer malignancy [10]. In the present results, we were unable to show any difference in plasma, HDL or non-HDL apoE concentration between patients with high proliferative index tumors when compared with patients with low proliferative index tumors. However, when considering the relative enrichment of HDL and non-HDL in apoE, it was found that RH+ patients with high proliferative index tumors exhibit non-HDL lipoprotein particles enriched in apoE, as suggested by the significantly higher apoE-to-apoB100 ratio in this group of patients.

Several isoforms of apoE may be found in Humans. The major isoforms are apoE2, apoE3 and apoE4, leading to six phenotypes, depending on the inherited ε alleles. There are some studies in the literature suggesting a link between the presence of the ε4 allele and breast cancer [33,34,35]. However, a meta-analysis found a significant relationship between the presence of this allele and breast cancer only in the Asian population [11]. The ApoE phenotype influences HDL and non-HDL levels in plasma and the apoE concentration [36]. The apoE distribution between HDL and non-HDL may vary with the ε alleles, and different apoE phenotypes distribute differently between lipoproteins [37]. Therefore, the relative enrichment of non-HDL that we observed in RH+ patients could be related to a different distribution of ε alleles between patients with high proliferative index tumors and patients with low proliferative index tumors. However, in our population, most of the patients carried the E3/E3 phenotype, and the ε allele distribution did not differ between groups of patients defined by their hormone receptor status and their proliferative index. Therefore, although we cannot exclude an influence of the apoE polymorphism, it does not explain why non-HDL were relatively enriched in apoE in RH+ patients with high proliferative index tumors.

This descriptive study shows that the distribution of apolipoproteins C-I, C-II, C-III and D between HDL and non-HDL is linked with the severity of breast cancer, as assessed by Ki-67.

## Figures and Tables

**Table 1:** Clinicobiological parameters of the studied cohort. Data are presented with median (25th–75th percentile) and frequencies as number (precentage).

	HR−			HR+		
	Ki-67 ≤ 20% (*n* = 19)	Ki-67 ≥ 30% (*n* = 29)	*p*	Ki-67 ≤ 20% (*n* = 51)	Ki-67 ≥ 30% (*n* = 41)	*p*
Age (years)	62.5 [56.0–69.0]	54.4 [43.0–66.0]	0.08	64.1 [56.0–72.5]	56.2 [48–66.0]	0.003
BMI (Kg/m^2^)	24.7 [21.8–27.3]	25.2 [22.2–27.1]	0.66	25.4 [22.7–27.5]	26.1 [21.9–28.8]	0.67
Menopause			0.15			0.006
Yes	17 (89%)	21 (72%)		43 (84.3%)	24 (58.8%)	
No	2 (11%)	8 (28%)		8 (15.7%)	17 (41.2%)	
Normolipidemic Treatment			0.85			0.71
Yes	3 (16%)	4 (14%)		5 (9.8%)	5 (12%)	
No	16 (84%)	25 (86%)		46 (90.2%)	36 (88%)	
Type of cancer			0.29			0.01
Inflitrating duct carcinoma	16 (84%)	28 (97%)		51 (100%)	36 (88%)	
Invasive lobular carcinoma	2 (11%)	1 (3%)		0 (0%)	5 (12%)	
Histopronostic grade			<0.001			<0.001
Grade I	0 (0%)	0 (0%)		21 (41.2%)	0 (0%)	
Grade II	17 (89%)	3 (10.3%)		26 (51%)	6 (14.6%)	
Grade III	2 (11%)	26 (89.7%)		4 (7.8%)	35 (85.4%)	
Molecular sub-types						
Luminal B HER2+				5 (9.8%)	15 (36.6%)	
Luminal B HER2−				0 (0%)	26 (63.4%)	<0.001
Luminal A				46 (90.2%)	0 (0%)	
TNBC	16 (84.2%)	21 (72.4%)	0.488			
HER2 Type	3 (15.8%)	8 (27.6%)				

**Table 2:** Plasma and lipoprotein lipids in estrogen receptor-positive (HR+) and negative (HR−) breast cancer patients, according to the proliferation index (Ki-67 ≤ 20% or Ki-67 ≥ 30%). Data are presented with median (25th–75th percentile).

	HR−			HR+		
Parameters	Ki-67 ≤ 20% (*n* = 19)	Ki-67 ≥ 30% (*n* = 29)	*p*	Ki-67 ≤ 20% (*n* = 51)	Ki-67 ≥ 30% (*n* = 41)	*p*
Plasma Cholesterol (mmol/L)	5.30 [4.53–5.93]	5.25 [4.07–5.75]	0.770	5.03 [4.17–5.83]	5.36 [4.44–5.91]	0.297
Plasma Triglycerides (mmol/L)	1.23 [0.91–1.74]	1.11 [0.79–1.37]	0.381	0.90 [0.71–1.25]	0.92 [0.74–1.12]	0.835
LDL Cholesterol (mmol/L	3.30 [2.47–3.82]	3.39 [2.39–4.01]	0.770	3.18 [2.49–3.90]	3.38 [2.60–3.97]	0.297
HDL Cholesterol (mmol/L)	1.48 [1.10–1.66]	1.39 [1.07–1.51]	0.144	1.30 [1.09–1.62]	1.38 [1.14–1.57]	0.297
Non HDL Cholesterol (mmol/L)	3.90 [3.19–4.65]	3.79 [2.82–4.46]	0.770	3.57 [2.94–4.39]	3.84 [3.17–4.50]	0.531

**Table 3:** Concentration of plasma, HDL and non-HDL apolipoproteins in estrogen receptor-positive (HR+) and negative (HR−) breast cancer patients, according to the proliferation index (Ki-67 ≤ 20% or Ki-67 ≥ 30%). Data are presented with median (25th–75th percentile).

	HR−			HR+		
Parameters	Ki-67 ≤ 20% (*n* = 19)	Ki-67 ≥ 30% (*n* = 29)	*p*	Ki-67 ≤ 20% (*n* = 51)	Ki-67 ≥ 30% (*n* = 41)	*p*
Plasma apoA-I (mg/dL)	148.60 [133.1–159.0]	160.0 [137.0–170.0]	0.381	152.40 [128.3–168.4]	148.84 [133.7–163.6]	0.531
Plasma apoB100 (mg/dL)	79.86 [68.3–104.4]	96.60 [78.1–117.0]	0.144	95.67 [76.5–118.8]	88.40 [75.9–108.8]	0.297
Plasma apoC-I (mg/dL)	2.15 [2.00–2.89]	2.46 [1.78–2.82]	0.381	1.99 [1.64–2.58]	2.30 [1.97–3.06]	0.060
HDL apoC-I (mg/dL)	1.61 [1.32–2.04]	1.34 [1.02–1.80]	0.041	1.35 [1.10–1.62]	1.56 [1.20–1.95]	0.022
Non-HDL apoC-I (mg/dL)	0.62 [0.11–1.29]	0.87 [0.43–1.37]	0.381	0.58 [0.26–1.07]	0.77 [0.43–0.98]	0.060
Plasma apoC-II (mg/dL)	1.97 [1.43–2.45]	2.00 [1.60–2.30]	0.770	2.00 [1.56–2.55]	2.00 [1.49–2.70]	0.835
HDL apoC-II (mg/dL)	1.31 [1.00–1.72]	1.48 [1.19–2.01]	0.381	1.67 [1.16–1.85]	1.73 [1.10–2.06]	0.531
Non-HDL apoC-II (mg/dL)	0.63 [0.39–1.02]	0.31 [0.18–0.65]	0.009	0.47 [ 0.17–0.74]	0.48 [0.23–0.71]	0.835
Plasma apoC-III (mg/dL)	4.54 [3.77–5.31]	4.20 [3.70–5.10]	0.144	4.49 [3.80–5.33]	4.94 [3.92–6.04]	0.297
HDL apoC-III (mg/dL)	2.66 [2.22–3.12]	2.60 [2.00–3.30]	0.381	2.38 [1.69–2.96]	2.80 [2.42–3.64]	0.022
Non-HDL apoC-III (mg/dL)	1.98 [1.31–3.13]	1.87 [1.07–2.71]	0.381	2.14 [1.29–2.95]	1.99 [1.40–2.74]	0.835
Plasma apoD (mg/dL)	3.07 [2.39–3.51]	3.24 [2.99–4.16]	0.042	3.61 [3.09–4.27]	3.40 [2.69–4.06]	0.233
HDL apoD (mg/dL)	2.45 [2.01–2.99]	2.74 [2.36–3.35]	0.035	2.88 [2.48–3.43]	2.63 [2.22–3.21]	0.156
Non-HDL apoD (mg/dL)	0.48 [0.38–0.65]	0.57 [0.37–0.69]	0.300	0.64 [0.51–0.91]	0.73 [0.49–0.96]	0.903
Plasma apoE (mg/dL)	6.07 [ 5.20–7.54]	5.71 [4.64–7.48]	0.770	6.11 [5.06–7.68]	6.30 [5.07–7.95]	0.835
HDL apoE (mg/dL)	2.75 [2.18–4.08]	2.99 [1.96–3.50]	0.144	2.80 [2.25–4.82]	3.32 [1.78–4.64]	0.297
Non-HDL apoE (mg/dL)	3.68 [2.31–4.42]	3.10 [2.40–4.70]	0.381	3.11 [1.90–4.23]	3.17 [2.63–3.96]	0.531

**Table 4:** Molar ratios between apolipoproteins and apoA-I in HDL or apoB100 in non-HDL. Data are presented with median (25th–75th percentile).

	HR−			HR+		
Molar Ratio	Ki-67 ≤20% (*n* = 19)	Ki-67 ≥30% (*n* = 29)	*p*	Ki-67 ≤20% (*n* = 51)	Ki-67 ≥30% (*n* = 41)	*p*
HDL apoC-I/apoA-I	0.045 [0.040–0.054]	0.030 [0.027–0.042]	0.001	0.031 [0.028–0.039]	0.039 [0.031–0.050]	0.007
Non-HDL apoC-I/ apoB100	0.56 [0.12–0.99]	0.64 [0.30–1.03]	0.381	0.46 [0.24–0.72]	0.64 [0.32–0.88]	0.144
HDL apoC-II/apoA-I	0.029 [0.024–0.037]	0.033 [0.027–0.036]	0.381	0.032 [0.027–0.038]	0.034 [0.025–0.042]	0.531
Non HDL apoC-II/ apoB100	0.51 [0.36–0.78]	0.21 [0.13–0.48]	0.001	0.28 [0.13–0.40]	0.34 [0.21–0.45]	0.531
HDL apoC-III/apoA-I	0.065 [0.055–0.074]	0.055 [0.046–0.066]	0.041	0.054 [0.038–0.063]	0.060 [0.052–0.084]	0.060
Non HDL apoC-III/ apoB100	1.44 [1.11–2.21]	1.15 [0.82–1.92]	0.381	1.32 [0.99–1.93]	1.47 [1.18–1.88]	0.297
HDL apoD/apoA-I	0.017 [0.015–0.021]	0.017 [0.016–0.019]	0.593	0.018 [0.015–0.024]	0.018 [0.015–0.021]	0.481
Non HDL apoD/ apoB100	0.14 [0.09–0.15]	0.11 [0.07–0.16]	0.821	0.12 [0.09–0.19]	0.16 [0.10–0.21]	0.537
HDL apoE/apoA-I	0.018 [0.014–0.023]	0.016 [0.010–0.019]	0.144	0.018 [0.012–0.023]	0.018 [0.013–0.023]	0.835
Non HDL apoE/ apoB100	0.66 [0.43–0.83]	0.51 [0.41–0.64]	0.144	0.48 [0.36–0.60]	0.58 [0.38–0.72]	0.022

## Data Availability

Data are available on request.
